# P-body-like condensates in the germline

**DOI:** 10.1016/j.semcdb.2023.06.010

**Published:** 2023-07-03

**Authors:** Madeline Cassani, Geraldine Seydoux

**Affiliations:** HHMI and Department of Molecular Biology and Genetics, Johns Hopkins University School of Medicine, Baltimore, MD 21205, USA

**Keywords:** P-body, Germline, Germ cells, Nanos, Germ granules, RNA

## Abstract

P-bodies are cytoplasmic condensates that accumulate low-translation mRNAs for temporary storage before translation or degradation. P-bodies have been best characterized in yeast and mammalian tissue culture cells. We describe here related condensates in the germline of animal models. Germline P-bodies have been reported at all stages of germline development from primordial germ cells to gametes. The activity of the universal germ cell fate regulator, Nanos, is linked to the mRNA decay function of P-bodies, and spatially-regulated condensation of P-body like condensates in embryos is required to localize mRNA regulators to primordial germ cells. In most cases, however, it is not known whether P-bodies represent functional compartments or non-functional condensation by-products that arise when ribonucleoprotein complexes saturate the cytoplasm. We speculate that the ubiquity of P-body-like condensates in germ cells reflects the strong reliance of the germline on cytoplasmic, rather than nuclear, mechanisms of gene regulation.

## 1. Introduction

Regulation of messenger RNAs (mRNAs) in the cytoplasm involves competition between two activities: translation and degradation. In a middle-ground purgatory, mRNAs are maintained in a silenced state, neither translated nor degraded, but stored until future conditions determine their fate. In eukaryotic cells, mRNAs in “purgatory” enrich in cytoplasmic condensates called “processing bodies” or “P-bodies” for short. P-bodies have traditionally been studied in cells grown in culture, such as yeast or mammalian cells, and are defined by the presence of a few conserved proteins (Table 1). In this review, we describe related condensates observed in the germline of animal models. During development, germ cells alternate between periods of high and low transcriptional activity and often rely on post-transcriptional mechanisms for gene regulation [72,90]. We first survey the different types of P-body-like granules reported in gametes (oocytes and sperm), embryonic germline progenitors, germline stem cells, and differentiating germ cells. We explore the possibility that the varying “flavors” of P-bodies arose as a consequence of stage-specific requirements for different classes of ribonucleoprotein (RNP) complexes. We discuss connections between the universal germ cell fate regulator Nanos and P-body RNPs. Finally, we discuss whether germline P-bodies are functional compartments or “incidental condensates”, non-essential condensation by-products that arise when sub-soluble RNP complexes saturate the cytoplasm.

### 1.1. What are P-bodies?

P-bodies were first described in mammalian tissue culture cells as microscopic puncta containing enzymes that remove mRNA caps (decapping factors 1 and 2, Dcp1/2) and degrade mRNAs in the 5′ to 3′ direction (Xrn1 exonuclease) [111,5]. Genetic analyses, primarily in yeast, indicated that P-bodies assemble around translationally-repressed, partially deadenylated mRNAs bound by distinct protein complexes at their 5′ and 3′ ends [91]. The cap-associated 5′ complex includes Dcp1/2 and their regulators (e.g. the enhancer of decapping Edc3) [32,98]. The 3′ complex includes Xrn1 and the scaffold protein Pat1 and the Lsm1–7 RING complex which recognizes short poly-A tails [107,14]. The 5′ and 3′ complexes interact with each other and with another essential P-body protein, the DEAD-box ATPase and translational repressor DDX6 (Dhh1p in yeast) [22,28,76], leading to a model in which the mRNA folds in a closed loop [27]. This configuration is thought to keep mRNAs out of the translational pool by blocking access to initiation factors (targeting the cap) and poly-A binding protein (targeting the poly-A tail), both of which are absent from P-bodies (reviewed in [27]). P-bodies have been proposed to form by liquid-liquid phase separation, a spontaneous process that causes multivalent complexes to de-mix from the cytoplasm to form dense condensates. In support of this view, seven P-body proteins have been reported to form co-condensates in vitro [23].

P-bodies were initially proposed to correspond to sites of mRNA decay [91] but subsequent studies showed that P-bodies are not essential for mRNA degradation, mRNA decay intermediates appear outside of P-bodies, and transcripts in P-bodies can exit P-bodies and become translated [9,16,34,44]. In a landmark study in 2017, Hubstenberger et al. purified P-bodies from a human epithelial cell line by fluorescence-activated particle sorting, and discovered that P-bodies enrich thousands of mRNAs, representing over a third of coding transcripts in that cell type [47]. P-body transcripts were no less abundant than other transcripts but were poorly translated, as evidenced by their low ribosome coverage and low protein yield. Remarkably, depletion of DDX6 by RNAi was sufficient to disassemble P-bodies and increase the translation rate of P-body-enriched transcripts in a manner proportional to their enrichment [47]. The prevailing hypothesis today is that P-bodies serve as temporary depos for translationally repressed, but translationally *competent*, mRNA molecules and their regulators. In this review, we consider related RNA granules that assemble in the germline of commonly studied animal models (Tables 1–3).

## 2. Gametes

### 2.1. P-body-like granules in oocytes are potential storage sites for translationally-repressed maternal mRNAs

**Fig. 1.**
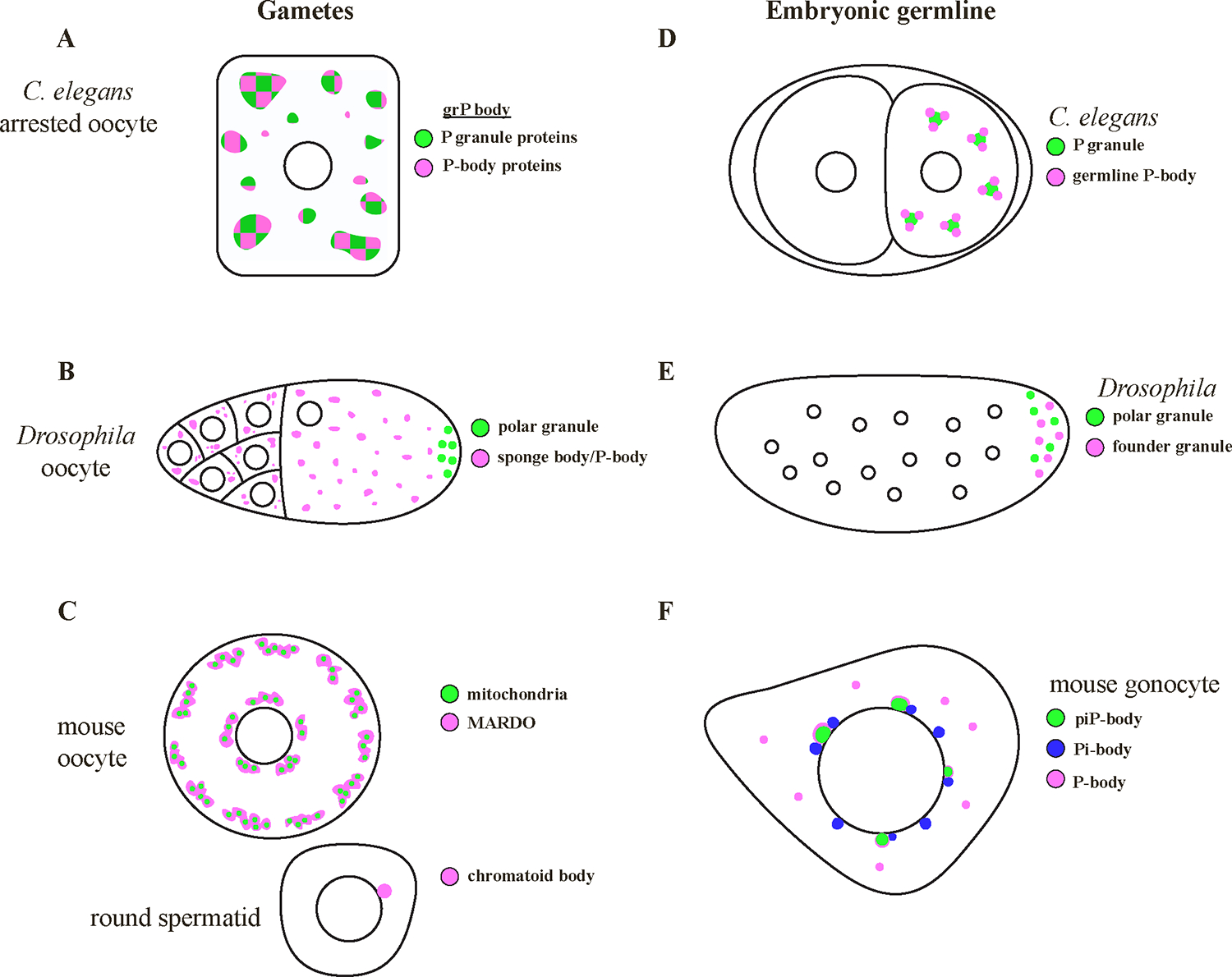
P-body like granules in gametes and embryonic germ cells. A) In *C. elegans*, oogenesis occurs in a syncytium, where germ cells progress through meiosis in an assembly-line like fashion. A large fraction of germ cells function as nurse cells and undergo apoptosis to provide RNA and protein to the surviving developing oocytes [80]. Large, stable grP bodies assemble in *C. elegans* oocytes that arrest when sperm is absent. These granules contain P granule components (green) and canonical P-body proteins (pink) that occupy distinct subdomains within the granule [46,52]. B) In *Drosophila*, oogenesis occurs in ovarioles, which consist of progressively developing egg chambers that are produced from the germarium, that contain the germline stem cells. Each egg chamber consists of 16 cells, including one oocyte and 15 nurse cells that provide RNA and protein for the oocyte [6]. In *Drosophila* egg chambers, sponge bodies/P-bodies (pink) form in the cytoplasm of nurse cells and the oocyte. Polar granules (green) localize to the posterior pole of the oocyte where the embryonic germline will form. C) MARDO (pink) assemble in mouse germinal vesicle (GV) stage oocytes and cluster around mitochondria (green). In round spermatids, the chromatid body (pink) associates with the nuclear membrane. Oocyte and round spermatid are not drawn to scale. D) In *C. elegans* early germline blastomeres, germline P-bodies (pink) enrich on the surface of P granules (green). E) In early *Drosophila* embryos, founder granules (pink) degrade *oskar* mRNA prior to pole cell formation. Polar granules (green) localize mRNAs required for pole cell development, such as Nanos, in the posterior. F) In mouse gonocytes, perinuclear foci termed piP-bodies (green) contain piRNA pathway proteins and canonical P-body components, which localize to the surface of the granule (pink). Pi-bodies (blue) containing MILI are distinct perinuclear granules that frequently localize adjacent to the piP-bodies. P-bodies containing Nanos2 and dead end1 also localize in the cytoplasm.

Oocytes synthesize many mRNAs for use during embryogenesis. These so-called “maternal mRNAs” are stored in granules that have been referred to by various names depending on the species (Table 1). Several oocyte granules contain canonical P-body proteins including the DEAD-box helicase DDX6, the translational repressor 4E-T, and the LSM-domain protein Lsm14 (Table 1 and references therein). Whereas yeast and tissue culture cell P-bodies are typically small (*<*1 μm) and uniform in size and composition, oocyte granules adopt various sizes and shapes, and contain different assortments of canonical P-body proteins (Table 1). For example, the grP bodies of aged *C. elegans* oocytes grow as large as 10 μm in size and segregate some components, such as the P-body protein Lsm14 (CAR-1), to distinct sub-granule domains ([13,46,52,77]; Fig. 1A). Likewise the sponge bodies of *Drosophila* oocytes vary in size and shape, from small dispersed puncta to larger reticulated bodies depending on developmental stage and environmental conditions ([125,95], Fig. 1B). In the pre-meiotic oocytes of mice, P-body proteins localize to a “mitochondria-associated ribonucleoprotein domain” (MARDO; previously described as subcortical aggregates by [19,36]; Table 1; Fig. 1C). MARDO consist of irregularly shaped granules which reach several microns in diameter and contain translationally repressed maternal mRNAs. Disruption of the MARDO leads to premature loss of MARDO localized mRNAs [19]. Similarly, DDX6 (Xp54) in *Xenopus* oocytes localizes to particulate structures throughout the cytoplasm as well as to the Balbiani body, a large RNA-rich aggregate that also contains mitochondria [58,94].

The function of oocyte granules has been tested by depleting oocytes of canonical P-body proteins, including DDX6. In *C. elegans*, loss of DDX6 (CGH-1) results in abnormally shaped grP bodies (as visualized by Lsm14), translational activation and destabilization of maternal mRNAs, and stunted oocyte development [13,12,38,4,74,77] In *Drosophila*, loss of DDX6 (Me31B) disrupts the translational repression of the maternal *oskar* and *bicD* mRNAs [73]. Furthermore, the P-body proteins 4E-T (Cup), Dcp1 (dDcp1), and Edc4 (dGe-1) contribute to the proper localization and/or stability of *oskar* in oocytes [123,17,35,62]. The DDX6 ortholog DOZI in plasmodium female gametocytes, and DDX6 and Lsm14 orthologues (Xp54 and xRAP55) in *Xenopus* oocytes have also been implicated in translational repression [105,58,65,70].

While these studies point to a requirement for P-body proteins in translational repression, an outstanding question is whether mRNA storage in P-bodies is required for, or merely a consequence of, translational repression. In *C. elegans* arrested oocytes, a *lacZ* reporter mRNA carrying the translationally repressed *glp-1* 3′UTR localized to grP-bodies and was not translated, while a *lacZ* mRNA lacking the *glp-1* 3′ UTR localized to the cytoplasm and was robustly translated [77]. In *Drosophila*, *grk* mRNA, which is translated in oocytes, enriches at the periphery of P-bodies along with translational activators, whereas *bcd* mRNA, which is repressed in oocytes, enriches in the interior of P-bodies. Following egg activation, *bcd* is translated and no longer colocalizes with P-bodies [119]. These experiments suggest a correlation between P-body localization and translational repression but do not demonstrate a causal effect. We return to this question in the concluding paragraphs of this review.

### 2.2. Dynamics of P-body-like granules increase during the oocyte-to-embryo transition

P-bodies in yeast and mammalian cells behave like liquid droplets [57] and assemble or disassemble in response to cellular conditions. For example, P-body assembly is enhanced by treatments that inhibit translation initiation, such as stress, and suppressed during mitosis and by treatments that block translation elongation [106,130]. Similarly, P-body dynamics change during the transition from the cell-cycle arrested, mostly translationally silent oocyte to the rapidly dividing, translationally active embryo. For example, in *C. elegans*, granule-to-cytoplasm exchange of GFP::CAR-1, as measured by fluorescence recovery after photobleaching (FRAP), increases by two orders of magnitude in embryos compared to oocytes [46]. In *Drosophila*, DDX6 (Me31B) dynamics also increase in embryos coincident with the release and translation of *bcd* mRNA [86]. Treatment with the aliphatic alcohol 1,6-hexanediol, which disrupts hydrophobic interactions, prematurely increases DDX6 dynamics and releases *bcd* mRNA [86].

In addition to changing dynamics, granules also change in their composition during the oocyte-to-embryo transition [119,13,63,67]. For instance, in stage 9 *Drosophila* oocytes, Dcp1-containing granules do not contain the Dcp1 partner and decapping enzyme Dcp2, nor the 5′ – 3′ exonuclease Xrn1 (Pacman) but acquire these components later in embryogenesis [62,63]. Biochemical and molecular evidence suggest that *Drosophila* DDX6 (Me31B) evolves from promoting translational repression in oocytes to promoting mRNA degradation in embryos [117]. Similarly, P-bodies recruit the decapping activators LSM-1 and LSM-3 coincident with the onset of maternal mRNA degradation in *C. elegans* embryos [38]. These data suggest the ribonucleoprotein (RNP) complexes in P-body-like condensates evolve from a storage function in oocytes to promoting RNA degradation and translation to meet the changing needs of developing embryos. Whether the changes in condensate dynamics are incidental to the changes in RNP composition or are functional and necessary to liberate mRNAs from a stored state remains to be determined.

### 2.3. The chromatoid body: a hub for post-transcriptional regulation of mRNAs in haploid sperm?

Unlike oocytes, sperm are not thought to transmit large quantities of mRNAs to support embryonic development. Developing spermatids, however, stop transcribing new mRNAs during genome compaction and thus rely on post-transcriptional mechanisms to regulate transcripts required for sperm differentiation [54,61]. The chromatoid body is a single, large RNA-rich granule found in the haploid spermatids of several vertebrates ([79,87]; Table 3; Fig. 1C). In mice, the chromatoid body develops from smaller granules in late pachytene spermatocytes that condense to form a single large granule by the round spermatid stage, the last transcriptionally active stage during spermatogenesis [54,61]. Several canonical P-body proteins and mRNA-binding proteins localize to the chromatoid body, implicating the chromatoid body as a primary site for post-transcriptional regulation [56,55]. The chromatoid body also contains components of the piRNA and miRNA machinery [56,55,68]. Interestingly, miRNA processing components have also been reported in the P-bodies of mammalian tissue culture cells [64,82,89]. The chromatoid body may correspond therefore to a specialized P-body that utilizes small RNAs for post-transcriptional mRNA regulation during spermatogenesis [2,54].

## 3. Embryonic germline

### 3.1. P-bodies are implicated in the specification of the embryonic germline

In some organisms, specification of the germ lineage depends on maternally inherited factors that enrich in germ plasm, a specialized cytoplasm that segregates with the embryonic germ lineage. Germ granules are condensates in germ plasm that enrich maternal mRNAs required for germ cell fate specification. Recently, a second class of condensates that contain P-body proteins has been described in the germ plasm of *Drosophila* and *C. elegans* ([18,33,42]; Table 2; Fig. 1D,E).

The germ granules of *Drosophila*, called polar granules, are assembled by the germ plasm organizer Oskar and contain several maternal mRNAs, including *Nanos*, that is translated and required in embryos for the development of “pole cells”, the progenitors of the germline [109]. In 2020, Eichler et al. described a second granule type in *Drosophila* germ plasm, called “founder granules”, that contain the P-body proteins DCP1, DDX6 (Me31B), and Xrn1 (Pacman) [33]. Founder granules accumulate and degrade maternal *osk* mRNA before pole cell budding to prevent it from interfering with pole cell development and migration to the gonad [33]. After pole cell budding, the polar granules themselves begin to accumulate mRNA degradation factors and degrade a subset of polar granule mRNAs, including *Nanos* [42]. Inactivation by RNAi of the decapping activators EDC3 and PATR-1 resulted in an increased number of pole cells that failed to migrate to the gonad [42]. These observations suggest that P-body-related activities are regulated in germ plasm to target specific maternal mRNAs at different developmental stages.

Similar observations were made recently in the germ plasm of *C. elegans* embryos. There, the germ granules that contain mRNAs essential for germline development, such as the Nanos homolog *nos-2*, are called P granules [99,101]. The canonical P-body proteins DDX6 (CGH-1) and EDC-3 assemble into distinct condensates (“germline P-bodies”) that exist either as independent granules in the cytoplasm or enriched on the surface of P granules ([18,38]; Table 2). Germline P-bodies exhibit complex patterns of localization before partially merging with P granules in the germline founder cell P_4_, coincident with activation of *Nanos* translation and turn-over of other maternal mRNAs. Two pairs of redundant, intrinsically-disordered proteins stabilize P granules (MEG-3 and MEG-4) and germline P-bodies (MEG-1 and MEG-2) in germ plasm [116,18]. Destabilization of P granules in *meg-3 meg-4* mutants prevents *nos-2* RNA assembly in granules and enrichment in P_4_, but surprisingly does not affect *nos-2* regulation. *meg-3 meg-4* embryos still repress *nos-2* translation early and activate *nos-2* translation in P_4_ and grow up into mostly fertile worms [60]. In contrast, failure to stabilize germline P-bodies in *meg-1 meg-2* mutants interferes with translation activation of *nos-2* and degradation of other maternal mRNAs, and leads to 100% sterile worms, where P_4_ descendants adopt mixed soma-like fates [18]. These observations suggest that, in *C. elegans* embryos, germ plasm condensates function primarily to concentrate mRNAs (P granules) and their regulators (germline P-bodies) for efficient delivery to the germline founder cell where they can operate in the cytoplasm. Localization of mRNAs inside the condensates, however, is not essential for mRNA regulation, nor is it sufficient to specify mRNA fate, since some germ granule mRNAs, such as Nanos, are translated and others are degraded in the germline founder cell.

### 3.2. Nanos, a P-body protein for the germline?

The broadly conserved *Nanos* family has been linked to germline development in a wide-range of organisms [26]. Animals typically have one or more Nanos homologs expressed at different stages of development, starting from the primordial germ cell stage. Nanos proteins contain tandem CCHC zinc fingers predicted to bind RNA and an N-terminal domain that recruits effector complexes that silence and/or degrade mRNAs [26]. The N-terminus of Nanos has been shown to interact with components of the CCR4-NOT deadenylase complex in mouse and *Drosophila* [103,53,85,8]. Deletion of the N-terminus prevents RNA degradation in vitro and prevents turnover of Nanos mRNA targets and germline development in mice [103,85,8].

Examination of NANOS2 in male germ cell progenitors (gonocytes) in mice was first to reveal Nanos enrichment in P-bodies ([102]; Table 2). DDX6 mutant germ cells in mouse chimeric embryos do not assemble P-bodies in gonocytes and maintain NANOS2 dispersed in the cytoplasm [93]. The NANOS 2 N-terminus is required for localization to P-bodies and recruitment of the CCR4-NOT complex member CNOT1 [103]. NANOS2 also interacts via its zinc finger domain with the RNA-binding protein Dead end (DND1), which also localizes to P-bodies and links NANOS2 to its mRNA targets [104,75]. Loss of DDX6 or DND1 phenocopies *Nanos2* mutants, including upregulation of target mRNAs. These observations suggest that NANOS2 requires P-body components to promote male germ cell development [93]. NANOS3, another mouse Nanos homolog required earlier in development in both sexes for primordial germ cell survival (Tsuda et al., 2003), also colocalizes with P-body components and interacts with the CCR4-NOT complex [129].

So far, localization of Nanos homologs in relation to P-bodies has not been extensively characterized in organisms outside of mice. The *Drosophila* CCR4 deadenylase colocalizes with Nanos in some foci in female germline stem cells [51], but Nanos localization with P-body components in other tissues has not been reported yet. Interestingly, loss of DND in zebrafish prevents efficient translation of Nanos on the surface of germ granules and causes germ cells to adopt somatic-like fates [121,41]. These phenotypes are reminiscent of those observed in *C. elegans* mutants that do not assemble germline P-bodies [18]. Transcriptional profiling of mutants that lack the redundant Nanos homologs *nos-1* and *nos-2* revealed that Nanos activity promote the degradation of hundreds of maternal mRNAs in *C. elegans* primordial germ cells [59]. Remarkably, lowering the maternal dose of *lin-15B*, a transcription factor that promotes somatic development, almost completely rescued the sterility of *nos-1 nos-2* mutants [59], suggesting that the primary role of Nanos in primordial germ cells is to eliminate mRNAs coding for soma-promoting factors. The emerging view is that Nanos activity throughout germ cell development is intimately linked to RNA silencing and decay promoted by factors associated with P-bodies. It will be important to investigate whether Nanos localization to P-bodies is conserved beyond mammals.

## 4. Stem cells and differentiating germ cells

### 4.1. A potential role for P-bodies in germline stem cell maintenance

In male gonads, the continuous production of gametes depends on spermatogonial stem cells (SSCs) that continually divide to both self-renew and produce cells that will differentiate into sperm. In mouse SSCs, NANOS2 colocalizes with DDX6 in P-body-like foci ([133], Table 3). Loss of NANOS2 or DDX6 in cultured germline stem cells lead to a stem cell maintenance defect and upregulation of differentiation genes, suggesting a defect in self-renewal [133]. NANOS2 co-immunoprecipitates with transcripts linked to differentiation and is required for their translational repression, association with DDX6, and enrichment in P-bodies [133].

Recently, DDX6 (Me31B) was reported to also contribute to stem cell homeostasis in the *Drosophila* testis but via a different mechanism [49]. In the *Drosophila* testis, stem cells are maintained by extracellular signals in the stem cell niche and also by dedifferentiation of spermatogonia called back by niche signals to replenish the stem cell pool [112,15]. Depletion of DDX6 caused an increased number of spermatogonia to dedifferentiate back into GSCs, likely due to a failure to repress *Nanos* translation in differentiating spermatogonia [49]. DDX6 had also been shown to contribute to Nanos translational repression in embryos [40,50].

Together, these studies suggest that DDX6 maintains tissue homeostasis using different mechanisms depending on cell context. In *Drosophila* spermatogonia, DDX6 promotes differentiation by inhibiting translation of *Nanos* mRNA, whereas in mouse germline stem cells, DDX6 and NANOS2 work together to maintain stem cell fate by preventing expression of transcripts involved in differentiation [133,49].

### 4.2. P-body connections to nuage and biosynthesis of small RNAs

Differentiating germ cells assemble perinuclear condensates (nuage) that enrich components of the small RNA amplification machinery that silence transposons and other foreign sequences [30]. Remarkably, P-body like condensates have been reported to associate with nuage in several systems. In mouse embryonic gonocytes, components of the piRNA pathway are segregated into two granules: Pi-bodies, which contain MILI and TDRD1, and piP-bodies, which contain MIWI2, TDRD9, and Maelstrom (MAEL), as well as canonical P-body proteins, some of which enrich at the surface of the granule ([3]; Table 2; Fig. 1F). MAEL is required for piRNA biogenesis and silencing of L1 transposons. In *Mael* mutants, P-bodies no longer associate with MIWI2 or TDRD9, suggesting that compartmentalization of piRNA pathway components into P-body like condensates is required for their activity [3]. piRNA pathway components were also recently reported to associate with P-bodies in adult mouse spermatocytes. At that stage, the piRNA ping pong amplification cycle is repressed, in part by the Tudor domain containing protein RNF17 and its interacting protein ADAD2 [118,127]. RNF17 and ADAD2 localize to P-bodies (identified by the P-body markers EDC3 and DCP1a). Upon knockout of *rnf17* or *adad2*, aberrant ping-pong occurs, leading to spermatogenesis arrest, and interestingly, a failure of P-bodies to assemble in diplotene spermatocytes [127]. In *C. elegans*, a recent preprint reports that P-body like condensates assemble at the periphery of nuage in meiotic germ cells and are required for small RNA homeostasis and transgenerational inheritance [31]. Together, these findings suggest a potential role for P-bodies in small RNA biogenesis and/or function in differentiating germ cells.

## 5. Conclusions and perspectives

As summarized in this review, studies in several animal models, from nematodes to vertebrates, have revealed that P-body like condensates are common in germ cells at all stages of development. Their varied appearance and composition suggest that germline P-bodies are flexible assemblies that accommodate a variety of RNP complexes with activities ranging from mRNA storage, translation and degradation to small RNA biogenesis and/or function. We suggest that the abundance of P-body-like condensates in germ cells reflects the heavy reliance of these cells on post-transcriptional mechanisms for gene regulation. Whereas somatic lineages depend on transcription factors for cell fate specification and differentiation, many key transitions in germline development are mediated by RNA-binding proteins that regulate mRNAs in the cytoplasm [90]. Most notably, as best described in mice, the universal germ cell fate regulator Nanos appears to function primarily by promoting mRNA degradation in cooperation with P-bodies.

An important question for the future will be to determine how P-body activities are modulated during developmental time to effect different outcomes, such as mRNA stability in oocytes and mRNA degradation in primordial germ cells, for example. Another question is whether germline P-bodies constitute functional compartments whose material properties facilitate RNA-focused activities not possible in the cytoplasm. For example, there is good evidence that localized condensation of germ granules and germline P-bodies in germ plasm has evolved as a mechanism to deliver high concentrations of mRNAs and their regulators to germline founder cells. It is also tempting to speculate that the low dynamics of oocyte granules may have evolved to protect mRNAs for long term storage away from the translational machinery, but this hypothesis remains untested. An alternative view is that some germline P-bodies may simply correspond to “incidental condensates”, non-essential condensation by-products of ribonucleoprotein complexes (RNPs) that saturate, and are active, in the cytoplasm [83]. Addressing this question will require quantitative and mutational analyses to distinguish a possible requirement for P-body assembly from a requirement for the RNPs that enrich in P-bodies. These types of analyses in yeast revealed that P-body proteins are more abundant in the cytoplasm than in P-bodies [126] and that mutants that suppress P-body condensation and maintain RNPs diffuse in the cytoplasm are still competent for RNA regulation [20,28,34], consistent with the incidental condensate hypothesis. Even if some germline P-bodies also turn out to correspond to incidental condensates rather than functional compartments, analysis of their assembly and composition may provide useful information as to the types of RNP complexes that support different stages of germ cell fate specification and differentiation. An important challenge for the future will be to understand how the two central enzymatic activities associated with P-bodies, RNA decapping and de-adenylation, cooperate to localize, silence, translate and degrade specific mRNAs throughout the life cycle of the germline. Single-molecule technologies that enable the visualization of RNA biochemistry in cells hold great promise to move the field forward [11,25].

## Figures and Tables

**Table 1:** P-body-like granules during oogenesis.

	Species	Protein components	citation

grP bodies in arrested oocytes	*C. elegans*	PGL-1, GLH-1, GLH-2, **MEX-1 (TTP)**, **MEX-3**	[88]
		PUF-5, **MEX-5 (TTP)**	[77]
		**DCAP-2**	[52,77]
		**CGH-1 (DDX6)**	[13,52,77]
		**CAR-1 (Lsm14)**	[52,77]
		PAB-1, TIA-1	[52]
		DCR-1	[7]
		MEG-3, PGL-3	[84]
sponge bodies/P-bodies	*Drosophila*	Exuperantia	[125]
		Yps	[124]
		**Me31B (DDX6)**	[73]
		Gus	[100]
		**Cup (4E-T)**, **eIf4E**, Btz	[123]
		**Trailerhitch (Lsm14)**	[122]
		**Dcp1**, **Dcp2**	[62]
		Dhc, BicD, Egl, Sqd	[29]
		**Pacman (Xrn1)**	[63]
		Hrb27C, Bru, **Orb (CPEB)**, **Staufen**	[96,95]
		BicC	[96]
		**dGe-1 (EDC4)**	[35]
subcortical aggregates/MARDO	mouse	**DDX6**, **CPEB**, YBX2, EIF4A3	[36]
		ZAR1, **LSM14B**, **4E-T**	[19]

Proteins in bold are homologs of human P-body proteins

**Table 2:** P-body-like granules in the embryonic germline.

	Species	Protein components	citation

founder granules	*Drosophila*	**Staufen**, **DCP1**, **Me31B (DDX6)**, **Pacman (Xrn1)**	[33]
germline P-bodies	*C. elegans*	**PATR-1**, **DCAP-1/2**, CCF-1 (CNOT7)*, **POS-1 (TTP)**, PAB-1, **CGH-1 (DDX6)**	[38]
		MEG-1, MEG-2, **EDC-3**	[18]
piP-bodies (male gonocytes)	mouse	MIWI2, TDRD9, MAEL, **GW182**, **DCP1a**, **DDX6**, **XRN1**	[3]
P-bodies (PGCs/male gonocytes)	mouse	Nanos2, **DCP1a**, **XRN1**, CNOT3*, **DDX6**	[102]
		Nanos3, TIAL1, p-EIF2A	[129]
		Dead end1	[104]

Proteins in bold are homologs of human P-body proteins

* Although some CCR4-NOT complex members enrich in P-bodies, others are cytoplasmic or their localization has not yet been described

**Table 3:** P-body-like granules during spermatogenesis.

	Species	Protein components	citation

P-body (spermatogonia)	*Drosophila*	**Pacman (Xrn1)**, **Dcp1**, **Me31B (DDX6)**	[131]
P-body (spermatogonial stem cells)	mouse	Nanos2, **DDX6**, **Dcp1a**	[133]
chromatoid body (sperm) †	mouse/rat/human	Actin	[113]
		snRNP Sm proteins	[10,71]
		Cytochrome c	[43]
		Histone H4	[120]
		p48, p52	[78]
		MVH	[108]
		MTR-1	[21]
		GRTH/Ddx25	[110]
		RanBPM	[92]
		MIWI, Dicer, **GW182**, **DCP1a**, **Ago2**, **Ago3**	[55]a
		KIF17b	[55]b
		TDRD1, TDRD6, TDRD7	[45]
		MAEL	[97]
		Mili	[115]
		GEMIN3, NANOS1, PUMILIO2	[39]
		TDRD5	[128]
		CLOCK, BMAL1	[81]
		SCaMC-1 L	[1]
		SAM68	[69]
		NSun2	[48]
		eIF4A3, RBM8A, **UPF1**, SMG1, SMG6	[68]
		CaMKIV	[114]
		FYCO1	[24]
		β-tubulin	[37]
		KSRP	[132]
		IP6K1	[66]

Proteins in bold are homologs of human P-body proteins

† 88 chromatoid body components identified by mass spectrometery [68] are not included in this table

## Data Availability

No data was used for the research described in the article.
